# Tuberculous Septic Arthritis of the Hip Following the Incision and Drainage of a Groin Abscess: A Case Report

**DOI:** 10.7759/cureus.54543

**Published:** 2024-02-20

**Authors:** Mahmood Alam, Robert J Okapa, Rola Husain

**Affiliations:** 1 Department of Orthopaedics, Salmaniya Medical Complex, Manama, BHR; 2 Department of Radiology, Salmaniya Medical Complex, Manama, BHR

**Keywords:** musculoskeletal tuberculosis, groin abscess, tuberculosis of the hip, septic arthritis, extrapulmonary tuberculosis (eptb)

## Abstract

Tuberculosis of the hip is a relatively rare type of septic arthritis that is seldom seen in the developed world today. While pyogenic septic arthritis may present with clear features that help in early diagnosis and treatment, many of these features are absent or overlap significantly with tuberculous arthritis, making the diagnosis a clinical challenge. Here, we present a case of tuberculous septic arthritis seen in our clinic following the surgical incision and drainage of a groin abscess with minimal hip symptoms. We discuss the therapeutic approach for the patient and briefly review other reported cases of tuberculous septic arthritis in the literature.

## Introduction

Septic arthritis of native joints is a relatively uncommon orthopedic disease with an estimated annual incidence of 1-35 cases per 100,000 individuals [1]. It represents a substantial challenge to orthopedic surgeons worldwide resulting in significant morbidity to patients if not managed promptly and appropriately [2,3]. Unlike its pyogenic counterpart, tuberculous septic arthritis is seldom seen in modern healthcare systems today, with most data documenting skeletal tuberculosis coming from the developing world. Tuberculosis of the hip is second to the spine in prevalence and constitutes around 15-20% of extrapulmonary musculoskeletal tuberculosis cases [4]. Pyogenic septic arthritis of the hip often presents with pain, fever, and systemic manifestations of an acutely inflamed joint. Tuberculosis of the hip can present with very similar features and often with significant overlap with its pyogenic counterpart, which often complicates and delays accurate diagnosis and treatment [4]. In some cases, tuberculosis of the hip may present with vague symptoms, without the presence of fever or systemic manifestations of tuberculosis, or even as a soft tissue mass mimicking a neoplastic process [5,6]. The significant variation in clinical manifestation and overlap with a plethora of orthopedic diseases presents a challenge to orthopedic surgeons in the assessment and treatment of this increasingly rare disease.

While medical treatment of tuberculous septic arthritis is well established, any delay in diagnosis and management, especially of its atypical features, may result in devastating sequelae to the patient [7]. Medical treatment with the established four-drug regimen of isoniazid, pyrazinamide, ethambutol, and rifampin is often successful in eradicating tuberculosis without the need for surgical intervention [4]. However, surgical intervention may be necessary in some cases to alleviate symptoms, avoid joint destruction, and as salvage for cases with advanced and established joint destruction [4,8]. Here, we present an insidious case of tuberculosis of the hip where the patient had minimal hip symptoms that started after the incision and drainage of a groin abscess.

## Case presentation

A 37-year-old Bahraini male with no prior history of medical illness presented to the outpatient clinic of a tertiary care center in the Kingdom of Bahrain for a routine follow-up of back and hip pain. He had undergone an incision and drainage of a groin abscess four months prior in another local institution. The abscess treatment course was complicated by Stevens-Johnson syndrome as a reaction to fluoroquinolone (ciprofloxacin) administered orally at that time. The patient was admitted to the medical ward at the time and received treatment from an attending dermatologist with subsequent improvement and discharge. When attending our clinic, the patient was off antibiotics, mobile with the support of crutches, and complained of mild but deep-seated right hip pain. He denied any fever, weight loss, and night sweats and did not experience any constitutional symptoms. He reported that he had long-standing back pain with radiculopathy that had been constantly increasing but denied any weakness, numbness, or paresthesia. He reported complete control of his bowel and bladder.

Upon physical examination, the patient was afebrile and comfortable with no distress. A local examination of the right hip revealed a small and shallow surgical wound at the medial aspect of the groin fold. The wound was around 3 cm in length and around 1 cm in depth at the site of the previous incision and drainage. The wound was healthy with pinkish granulation tissue at the base and no evidence of slough or discharge. A swelling was noted distal to the wound over the medial side of the thigh. It was firm, around 8 cm in diameter, non-tender, non-fluctuant, and not fixed to the skin or the underlying tissue.

The hip appeared normal on examination with no deformity, fully passive, and active range of motion. No point tenderness was elicited on palpation of bony prominences. Both extremities had palpable and symmetrical pulses, and a neurological examination was unremarkable.

MRI of the hip was ordered in a prior outpatient visit and showed suspicion of septic arthritis of the right hip with proximal femoral osteomyelitis. The patient was admitted to the hospital and underwent blood and imaging investigations. Urine, blood, sputum, and wound cultures were obtained (Appendix shows detailed numbers of inflammatory markers throughout the treatment course). The patient was then started on empiric therapy with meropenem and vancomycin after consulting the infectious disease specialists.

**Figure 1:**
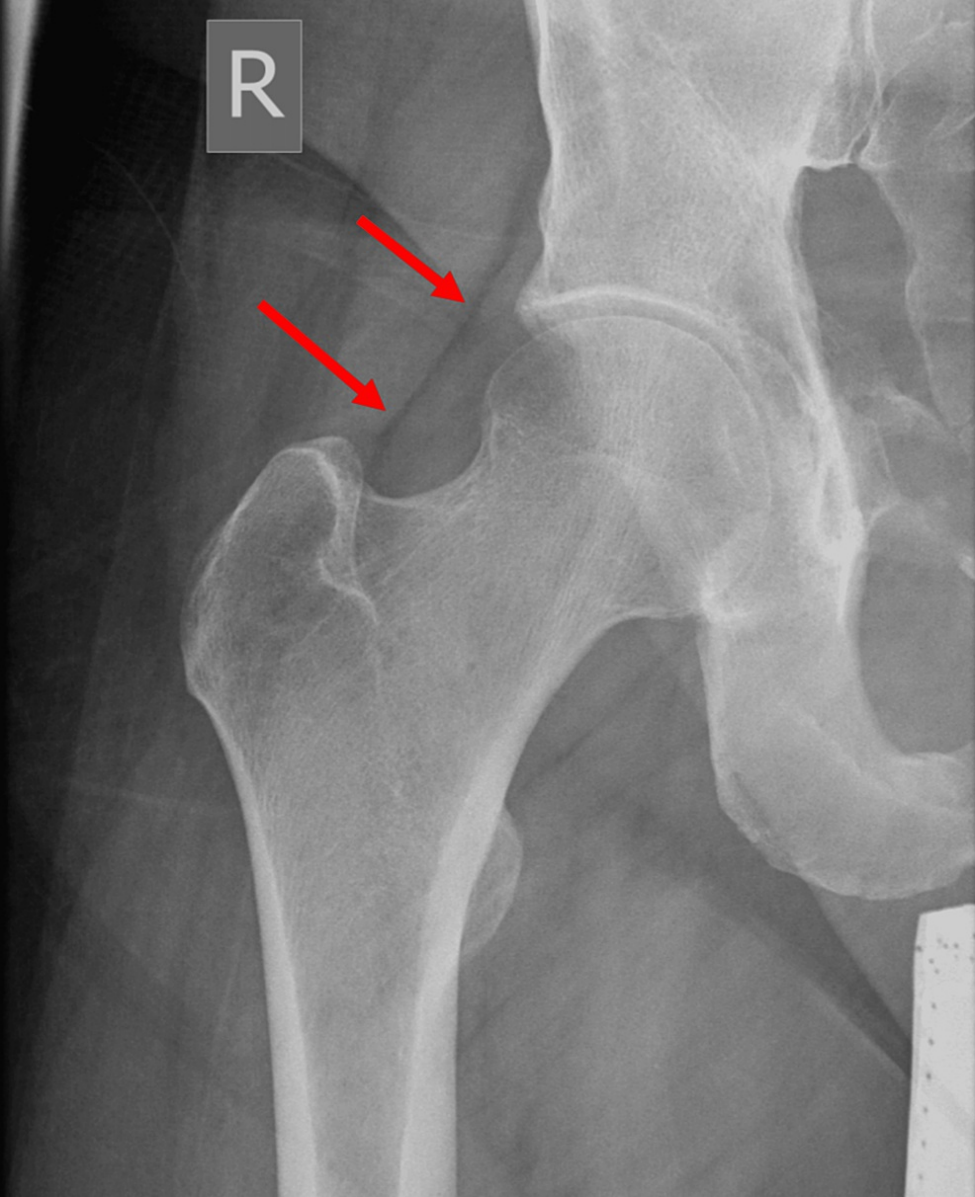
Radiograph of the right hip. Right-sided hip effusion (arrows).

**Figure 2:**
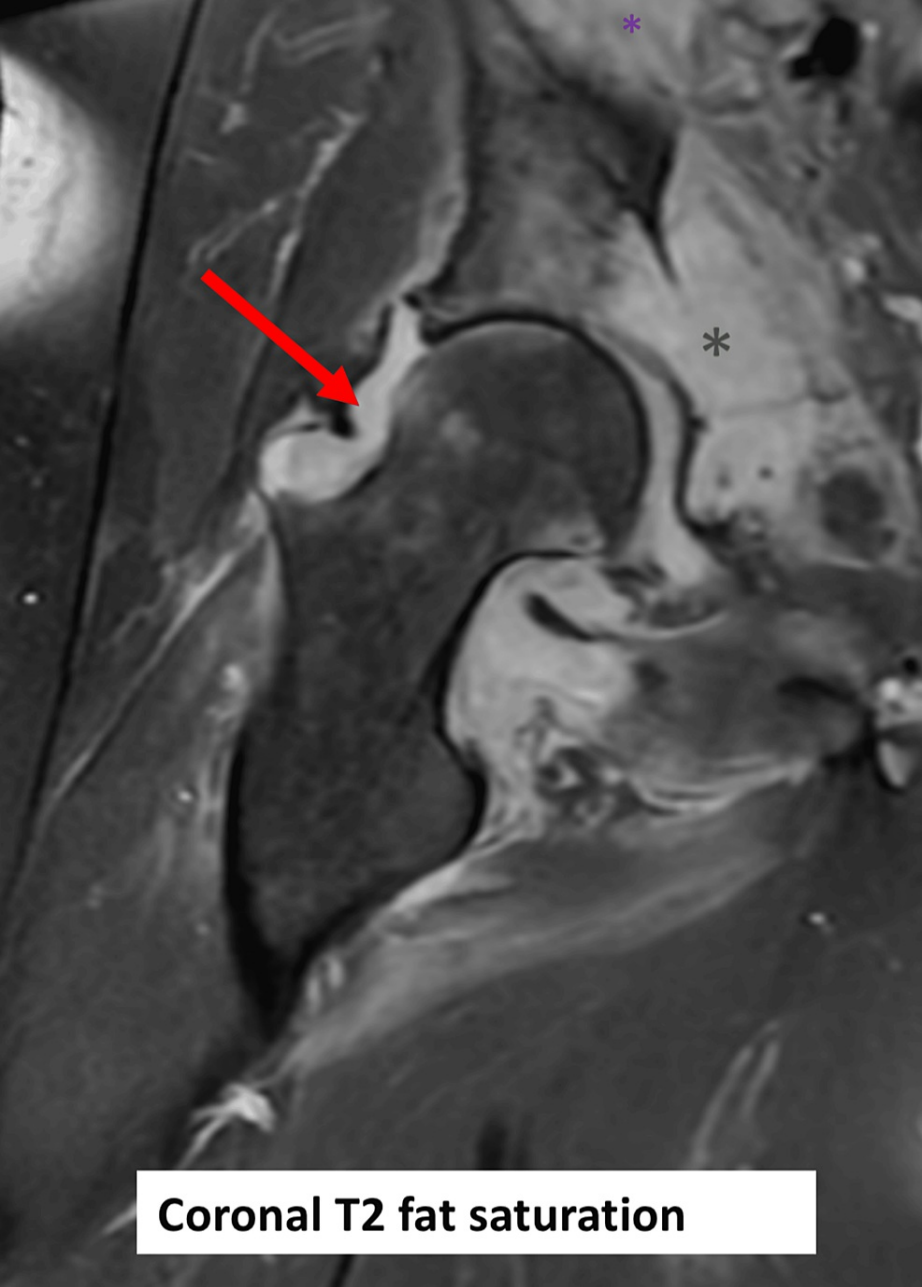
Right hip MRI (coronal T2 fat saturation). Right-sided joint effusion (red arrows) with acetabular destruction (green asterisk).

**Figure 3:**
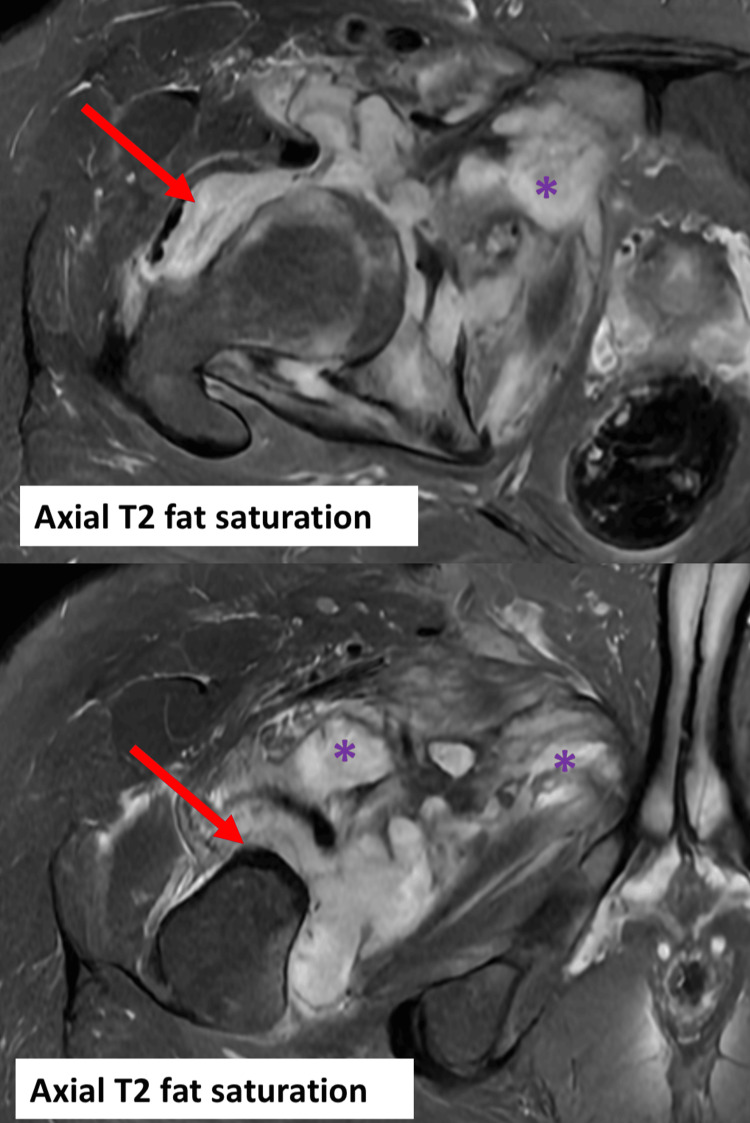
MRI of the right hip with axial T2 fat saturation. Right-sided joint effusion (red arrows) and multifocal intramuscular collections (purple asterisks) tracking into the medial thigh compartment and medial pelvis.

**Figure 4:**
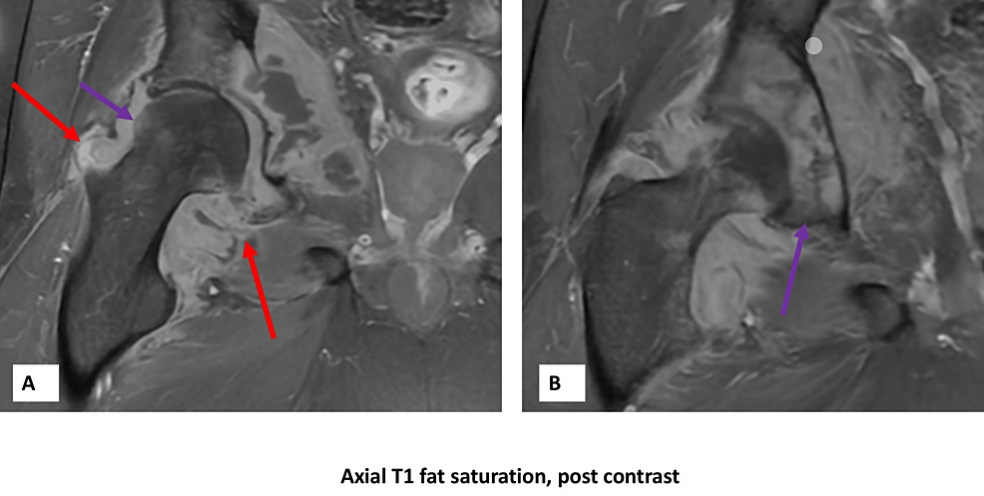
MRI of the right hip after contrast administration. (A) Enhancement of the joint fluid (red arrows), as well as the acetabulum (purple arrows), suggestive of septic arthritis and osteomyelitis, respectively. (B) Enhancement of around the acetabulum (purple arrow).

Plain imaging showed effusion of the right hip with a fat pad sign but was otherwise unremarkable (Figure 1). Initial MRI showed joint effusion with the erosion of the medial aspect of the femoral neck and acetabulum reflecting osteomyelitis and septic arthritis. Additionally, multifocal intramuscular collections tracking to the medial thigh were noted (Figures 2-4).

Laboratory tests preoperatively showed elevated inflammatory markers and normal white blood cell count. The patient was scheduled for surgical arthrotomy with debridement and drainage of septic arthritis as soon as he was medically fit. Operative arthrotomy was done utilizing the posterior approach to the hip. Intraoperative assessment revealed minimal changes to the bone with a completely healthy proximal femur and minimal joint collection of serous fluid with abundant whitish/gray cheesy material found in the hip joint with the majority of the collection concentrated around the anterior aspect of the hip. The consistency, character, and color of the biological material encountered during the arthrotomy raised suspicion of granulomatous inflammation. The tissue was sent for culture (including Lownstein-Jensen growth medium), gram stain, Ziehl-Neelsen staining, and histopathological analysis. A drain was inserted during the surgery and the wound was subsequently primarily closed.

Results of cultures obtained from the wound on admission returned *Pseudomonas aeruginosa* and methicillin-resistant *Staphylococcus aureus* (sensitive to meropanem and vancomycin). A six-week course of antibiotics was planned for the patient. Blood, urine, and sputum cultures obtained on admission did not show any bacterial growth. The patient was then followed up with serial inflammatory markers (twice weekly) and physical examination. The patient’s inflammatory markers increased after surgery and then progressively reduced but did not normalize fully postoperatively. The patient showed significant reported improvement in his hip pain postoperatively but he reported some minimal residual pain.

**Figure 5:**
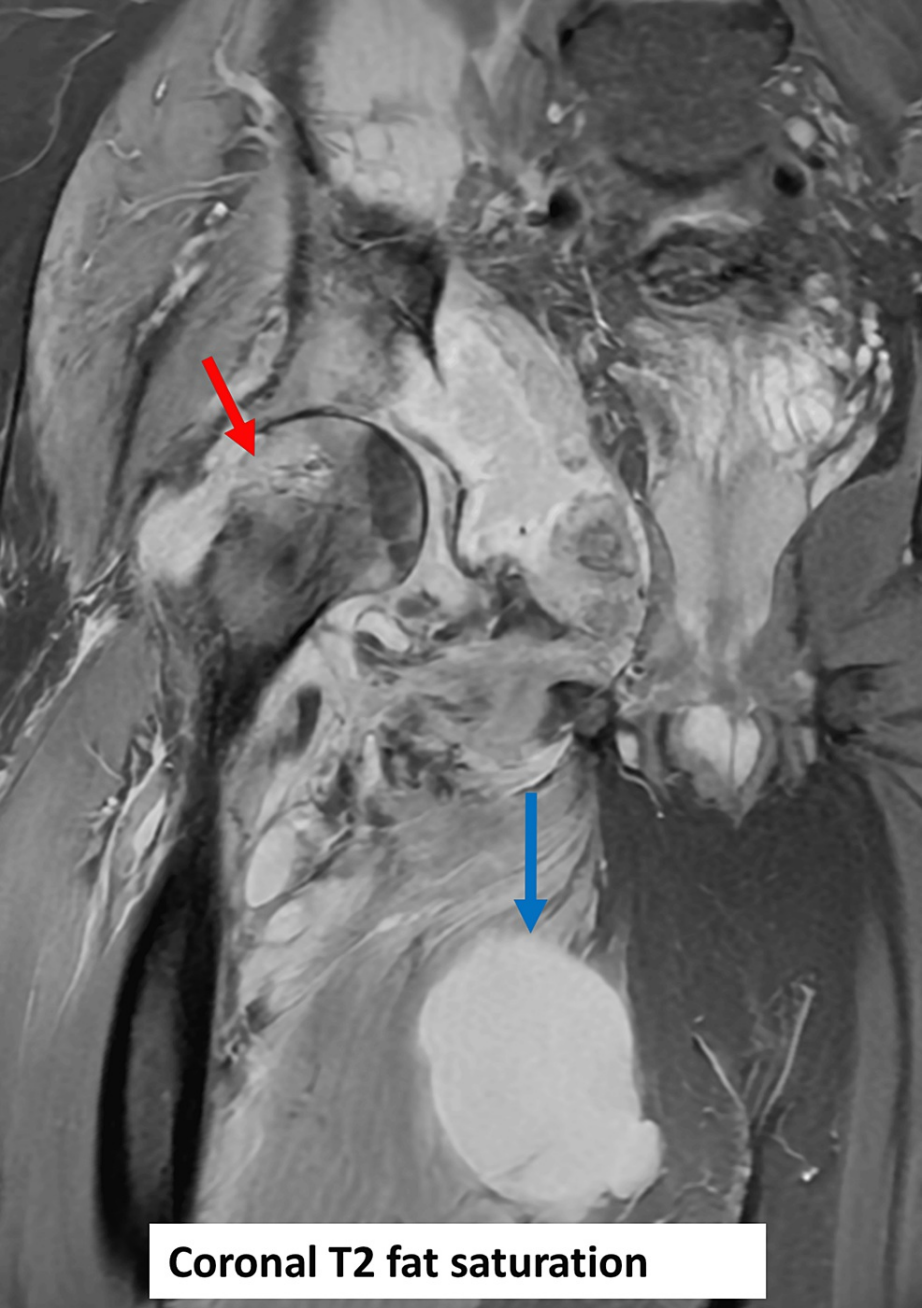
MRI of the right hip with coronal T2 fat saturation. Interval progression of osteomyelitis to the femoral head and neck (red arrow) with a grossly stable appearance of collections distribution with interval increase in the size of adductor collections (blue arrow).

**Figure 6:**
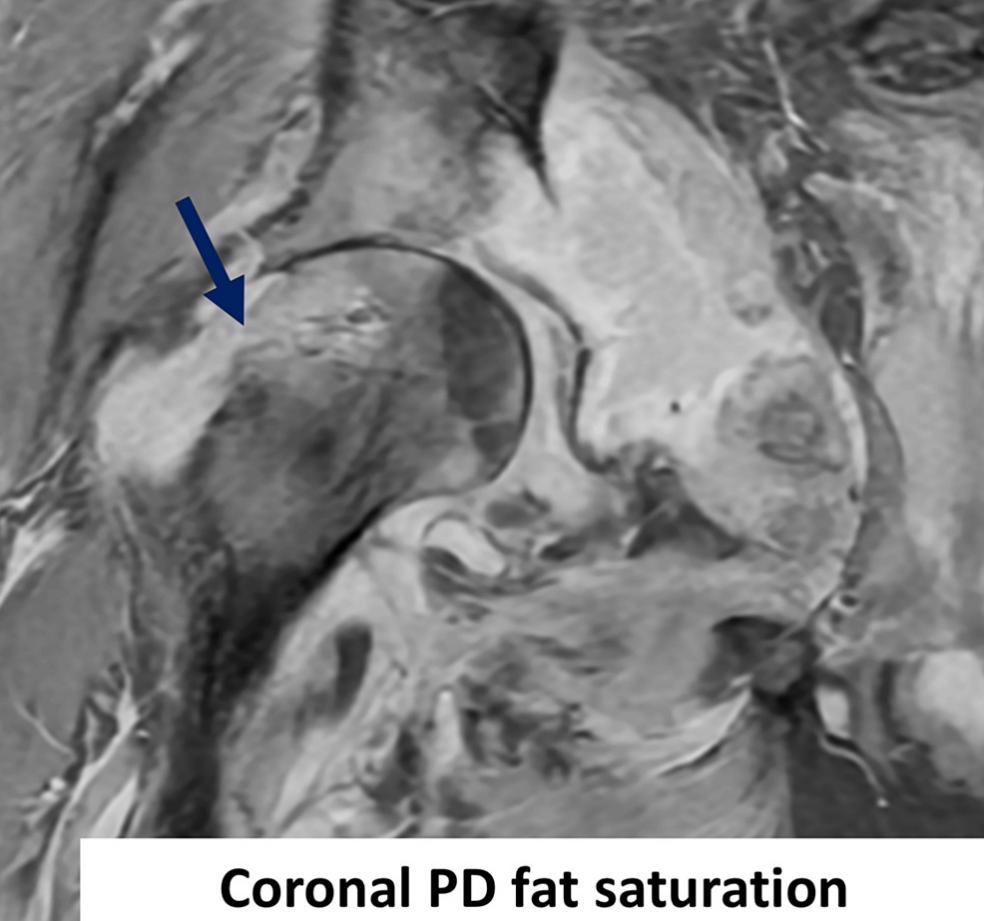
MRI of the right hip with coronal PD fat saturation. Interval development of erosive changes within the lateral aspect of the femoral head and neck (deep blue arrow). PD: proton density

Three weeks after the operative arthrotomy an MRI was ordered as part of the postoperative follow-up and showed interval reduction of the septic arthritis and progression of the osteomyelitis and medial thigh collection (Figures 5, 6). The findings of the follow-up MRI resulted in a decision of second surgical intervention in the form of incision and drainage of the medial collection. Drainage was performed jointly with general surgeons utilizing the medial approach to the thigh. Intraoperatively, around 100 mL of straw-colored fluid with whitish specs of tissue was drained. The fluid and tissue were sent for gram stain, Ziehl-Neelsen staining, and cultures (including the Lowenstein-Jensen growth medium). A surgical drain was placed and the wound was primarily closed.

Subsequently, the patient was planned to continue on the same antibiotics for six weeks as standard treatment of septic arthritis until further results were obtained. Partial weight bearing was allowed immediately postoperatively with the progression of weight bearing as tolerated. Moreover, the wounds were dressed daily in the ward.

The patient showed significant improvement after surgical drainage of the collections, with a near-complete resolution of the swelling and indolent pain that he was complaining of. Follow-up investigations revealed a sterile conventional culture, negative gram stain, and negative Ziehl-Neelsen stain for acid-fast bacilli. Histopathological analysis showed granulomatous inflammation with multiple necrotizing granulomas composed of epithelioid cells, Langerhans cells, and foreign body-type giant cells. The patient was then planned to continue his antibiotic (meropenem and vancomycin) treatment as a case of pyogenic septic arthritis pending further confirmation of a specific origin of arthritis. The patient was followed up with inflammatory markers which normalized one week postoperatively, and he was ambulatory and pain-free postoperatively.

**Figure 7:**
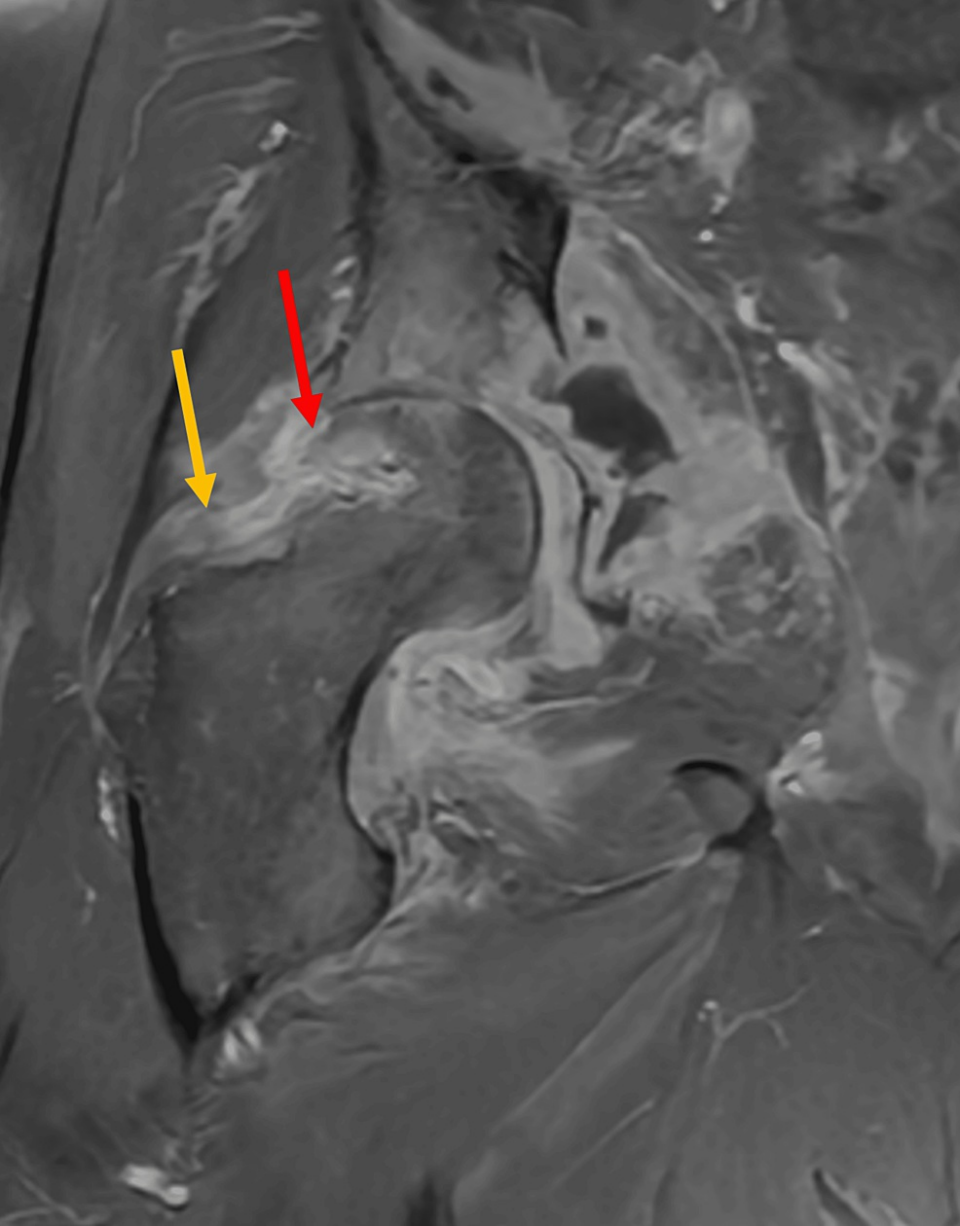
MRI of the right hip. Stable appearance of septic arthritis (yellow arrow). Interval regression of osteomyelitis to the femoral head and neck (red arrows). The grossly stable appearance of collections distribution with interval reduction in the number of adductor collections.

A follow-up MRI was ordered one week after the second surgery and showed a reduction of the medial collection and interval regression of septic arthritis (Figure 7), but revealed a new collection that was traced down from the superior iliopsoas region to the groin wound near the site of the previous incision and drainage. The general surgeon was called to assess the patient, who recommended percutaneous drainage of the iliopsoas collection under ultrasound guidance which was performed by an interventional radiologist. Stains (both for bacteria and mycobacteria) and cultures of the fluid were sent. The percutaneous drain placed by the interventional radiologist was retained for two days after the insertion during which it collected small amounts of whitish fluid. The percutaneous drain was subsequently removed when it stopped draining fluid two days after its insertion.

Three weeks after the operative drainage of the medial thigh collection (second surgery), the public health department notified the treating team that cultures of tissue collected during the surgical arthrotomy grew *Mycobacterium tuberculosis*. The infectious disease department was then involved, and they recommended a six-month anti-tuberculous treatment course which was initiated. The recommended regimen comprised isoniazid, pyrazinamide, ethambutol, and rifampicin. The patient is currently being followed up on an outpatient basis with significant improvement in hip symptoms and his wounds are healing satisfactorily.

## Discussion

Tuberculosis of the musculoskeletal system may present with various clinical manifestations that may not closely resemble a typical septic joint [5,6]. While it is important to consider this diagnosis in frail and immunocompromised patients, it is crucial to keep it in the differential diagnosis of healthy patients with no clear predisposing cause, especially when conventional treatment fails to achieve a satisfactory outcome.

While tuberculosis of the hip often presents with typical hip pain, clinicians should be vigilant when assessing cases of atypical hip pain to avoid missing important but rare diagnoses [4]. This is further emphasized by the lack of systemic or pulmonary manifestation of tuberculosis in our case. Orthopedic surgeons should always consider tuberculosis when assessing cases that are slowly progressing, those with indolent and atypical symptoms, and those with recurrent visits or failure to respond to conventional therapy. Delay in diagnosis is not infrequent in such cases with some reporting delay of up to two months before definitive diagnosis of tuberculous arthritis [9]. This is reinforced by our case where all stains were negative, and the slow-growing nature of the organism led to six weeks of delay in the final diagnosis until the results of the culture were received by the primary treating team.

The importance of a multidisciplinary approach with close communication between orthopedic surgeons, radiologists, pathologists, and infectious disease specialists is paramount in achieving good outcomes for patients with these complex and atypical presentations. A thorough assessment of MRI and plain imaging may provide very useful information to aid in the diagnosis of such cases. Some studies have pointed out salient features that may help flag cases of tuberculous arthritis and differentiate them from typical pyogenic arthritis [10,11]. MRI findings such as a disproportionate increase in bone erosions, chronic pannus formation, bone chips, and hypointense synovium favor tuberculous arthritis over its pyogenic counterpart [11].

Our review of published literature showed a few case reports of tuberculosis of the hip joint, each with varying manifestations and peculiarities. While some cases such as that reported by Al-Tikrity et al. showed more or less the typical form of primary tuberculosis with most of the salient features of tuberculosis of the hip such as pain, limitation of motion, and MRI features suggestive of tuberculosis [5], other cases such as the one published by Alsaleem et al. showed a more insidious course with little or no manifestation of an acutely septic joint and with MRI features that are not specific to tuberculous arthritis [12]. Other studies reported a more unique presentation of hip tuberculous arthritis that resembled a soft tissue tumor [6]. This wide spectrum of manifestations presents a unique challenge to orthopedic surgeons and stresses the importance of a high index of suspicion when managing cases of septic arthritis of the hip.

Treatment of tuberculous arthritis of the hip should be initiated as soon as the diagnosis is confirmed. Early communication with infectious disease specialists and the public health department is encouraged to provide the best outcome. The standard four-drug regimen is often recommended and accepted for all stages and presentation of the disease. The duration of therapy is variable between 6 and 12 months depending on the extent and severity of the disease and its manifestations [13]. We settled our patient on a six-month course of oral pharmacotherapy based on the recommendation of our local infectious disease department. While our case had little joint erosion and destruction, other cases reported extensive bone damage that necessitated resection or total hip arthroplasty. We recommend counseling the patient about possible long-term sequelae of such infections to set appropriate expectations for the future [8].

## Conclusions

Tuberculosis of the hip joint is an uncommon orthopedic manifestation of extrapulmonary tuberculosis that often presents with variable clinical signs making diagnosis challenging. Orthopedic surgeons should maintain a high index of suspicion when facing atypical presentation of septic arthritis or when conventional therapy fails to achieve a satisfactory result. A multidisciplinary approach with close communication between orthopedic surgeons, radiologists, and infectious disease specialists is crucial to achieve optimal outcomes for the patient.

## Appendices

**Table 1:** Timeline of inflammatory markers. ESR: erythrocyte sedimentation rate; CRP: C-reactive protein

Week	ESR	CRP	Comment
First week	95	74	MRI showed suspicion of septic arthritis
Second week	120	216	Postoperative from hip arthrotomy
Third week	90	20.9	Interval regression of septic arthritis and progression of osteomyelitis and medial collection. Medial incision and drainage were done
Fourth week	72	14.7	Post-medial collection incision and drainage. MRI showed iliopsoas collection
Fifth week	50	19.4	Post-percutaneous drainage of iliopsoas collection
Sixth week	24	9.58	One week post-drainage of all collections
